# Peripheral BDNF and psycho-behavioral aspects are positively modulated by high-intensity intermittent exercise and fitness in healthy women

**DOI:** 10.1038/s41598-021-83072-9

**Published:** 2021-02-18

**Authors:** Rodrigo Araujo Bonetti de Poli, Vithor Hugo Fialho Lopes, Fábio Santos Lira, Alessandro Moura Zagatto, Alberto Jimenez-Maldonado, Barbara Moura Antunes

**Affiliations:** 1 Laboratory of Physiology and Sport Performance (LAFIDE), Post-Graduate Program in Movement Sciences, Department of Physical Education, School of Sciences, São Paulo State University (UNESP), Av. Eng. Luiz Edmundo Carrijo Coube, 14-01, Vargem LimpaBauru, SP CEP 17033-360 Brazil; 2 Exercise and Immunometabolism Research Group, Post-Graduation Program in Movement Sciences, Department of Physical Education, São Paulo State University (UNESP), Presidente Prudente, SP Brazil; 3 Facultad de Deportes Campus Ensenada, Universidad Autónoma de Baja California, Ensenada, Mexico

**Keywords:** Metabolism, Cytokines, Neuroimmunology

## Abstract

Acute high-intensity intermittent exercise (HIIE) induces the myokine secretion associated with neurogenesis, as well brain-derived neurotrophic factor (BDNF); however, it remains unknown how the menstrual phase influences this secretion after an acute exercise session. The current study aimed to investigate the effects of HIIE performed in luteal and follicular menstrual phases on BDNF, cognitive function, mood, and exercise enjoyment. Fourteen healthy women completed four experimental sessions, randomly. One graded exercise test (GXT) and one HIIE session (10 × 1-min runs 90% peak GXT velocity [1-min recovery]) were performed for each menstrual phase. Blood samples were collected at rest and immediately after efforts, and the profile of mood states questionnaire (POMS) and Stroop-task test were applied. During the HIIE, subjective scales were applied (feeling, felt arousal, rate of perceived exertion, and physical activity enjoyment). The main results showed that the serum BDNF presented no difference between menstrual phases (*p* = 0.870); however, HIIE increased BDNF concentration in both menstrual phases (*p* = 0.030). In addition, the magnitude of circulating BDNF variation (Δ%BDNF) and $\dot{\text{V}}{{\text{O}}}_{\text{2max}}$ demonstrated an inverse relationship in the follicular phase (r =  − 0.539, *p* = 0.046), whereas in the luteal phase, Δ%BDNF was negatively correlated with time test (r =  − 0.684, *p* = 0.007) and RPE (r =  − 0.726, *p* = 0.004) in GXT. No differences between menstrual phases were observed for POMS (*p* ≥ 0.05); however, HIIE attenuated tension (*p* < 0.01), depression (*p* < 0.01), and anger moods (*p* < 0.01), independently of menstrual phases. The subjective scales and Stroop-task test did not show differences. In conclusion, menstrual cycle phase does not affect serum BDNF levels, cognitive function, mood, and exercise enjoyment. Contrary, HIIE increases peripheral BDNF and attenuates tension, depression, and anger independently of menstrual phase. In addition, Δ%BDNF was correlated with physical fitness in the follicular phase, exhibiting higher changes in women with lower physical fitness status.

## Introduction

Physical exercise activates several metabolic pathways in skeletal muscle, and regulates the synthesis and secretion of molecules known as myokines^1–4^. Myokines have autocrine, paracrine, or endocrine effects^5^. Therefore, through myokines, skeletal muscle generates crosstalk with other organs such as adipose tissue, liver, and brain^6,7^. In detail, Cathepsin-B and Irisin were identified as myokines that enhance the brain-derived neurotrophic factor (BDNF) expression in the brain^8^, an effect that was found even after a single bout of high-intensity intermittent exercise (HIIE)^8–10^. In agreement with this, other authors reported higher peripheral BDNF levels after HIIE^11^. BDNF is a neurotrophin that regulates molecular pathways associated with neurogenesis, synaptic plasticity, and metabolism in neurons^12^. Prior information emphasizes the potential of intermittent exercise modalities to promote brain health, since BDNF can induce growth and proliferation of cells in the hippocampus, influencing cognitive functions (i.e., memory and learning)^13,14^. In fact, it is known that physical exercise also arouses dopamine pathways and, consequently, acts on neurobehavioral systems, positively influencing mood, exercise enjoyment, and perceived exertion^15^.

However, besides physical exercise, other factors can modify BDNF concentrations, such as ovarian hormones^16^. The menstrual cycle generally occurs over ~ 28 days and is divided into three phases (i.e., follicular phase, ovulation, and luteal phase). Mainly during follicular and luteal phases, many fluctuations in ovarian hormones (i.e., estrogen and progesterone) occur for maturation of follicles and destruction of the corpus luteum to start a new cycle^17^. These fluctuations in ovarian hormones may directly impact the immune system^18^ and resting BDNF release^19^. In light of this, Begliuomini et al.^19^ analyzed BDNF kinetics during the menstrual cycle in specific populations (ovulatory, amenorrhoeic, and postmenopausal women) and reported higher BDNF concentrations during the luteal phase than follicular phase; the same study found a positive relationship between BDNF and hormone concentrations expressed during the luteal phase (i.e., progesterone). These data suggest that the menstrual cycle (i.e., hormonal variation) directly impacts the brain (i.e., central nervous system) modifying BDNF concentrations. In fact, it was previously reported that ovarian hormones alter different brain functions such as cognition, memory, and mood^20^.

Although previous studies have demonstrated the influence of the menstrual cycle on resting BDNF, its impact on HIIE responses and the relationship between physical performance and BDNF remain unclear. Therefore, the principal aim of the current study was to investigate the influence of luteal and follicular menstrual cycle phases on exercise-induced BDNF concentrations, cognitive function, mood, and exercise enjoyment in healthy women. Considering that menstrual cycle may influence resting levels of circulating BDNF (i.e., higher concentrations during luteal than follicular phase)^16^, it was hypothesized that HIIE would induce acute higher BDNF concentrations and greater cognitive function, mood (i.e., more vigor and less fatigue levels), and exercise enjoyment in the exercise session performed during the luteal phase compared to the follicular phase.

## Results

The parameters measured during the GXTs and comparisons between luteal and follicular phases are shown in Supplemental Table S1. No differences were observed for any parameters measured during the GXTs between menstrual phases (*p* ≥ 0.13).

Regarding the results of the mood states profile measured prior to and immediately after HIIE (Table 1), no significant interactions among time and conditions or condition effects were observed for any POMS dimensions. In contrast, HIIE significantly mitigated (i.e., ANOVA time effect) tension (*p* = 0.003; *F* = 14.355; *ƞ*^2^ = 0.545), depression (*p* = 0.008; *F* = 10.222; *ƞ*^*2*^ = 0.460), and anger (*p* = 0.001; *F* = 18.932; *ƞ*^*2*^ = 0.612) moods. Enjoyment measured by PACES was not different between luteal and follicular phases [68.07 ± 5.90 arbitrary unit (a.u.) (CI95% 64.66 to 71.48 a.u.) and 67.36 ± 6.00 a.u. (CI95% 63.90 to 70.82 a.u.), respectively; *p* = 0.72].

**Table 1:** Dimensions of mood measured by POMS.

Luteal phase		Follicular phase		ANOVA *p* (*F*)		
				Interaction (time × condition)	Time effect	Condition effect
Pre HIIE	Post HIIE	Pre HIIE	Post HIIE			
**Tension** ^#^*						
12.77 ± 6.22 (9.01 to 16.53)	10.54 ± 6.94 (6.35 to 14.73)	10.54 ± 2.96 (8.75 to 12.33)	7.38 ± 4.11 (4.90 to 9.87)	0.323 (1.064)	0.003 (14.355)	0.052 (4.639)
**Depression**^#^						
8.00 ± 10.85 (1.44 to 14.56)	5.23 ± 7.17 (0.90 to 9.56)	4.00 ± 2.80 (2.31 to 5.69)	2.15 ± 2.51 (0.64 to 3.67)	0.595 (0.297)	0.008 (10.222)	0.145 (2.433)
**Anger**						
7.31 ± 8.06 (2.44 to 12.18)	5.62 ± 7.19 (1.27 to 9.96)	5.54 ± 6.37 (1.69 to 9.39)	2.23 ± 3.79 (− 0.06 to 4.52)	0.428 (0.673)	0.001 (18.932)	0.188 (1.949)
**Vigor**						
14.46 ± 7.83 (9.73 to 19.19)	15.69 ± 8.04 (10.84 to 10.55)	16.92 ± 6.66 (12.90 to 20.95)	18.23 ± 6.80 (14.12 to 22.34)	0.954 (0.003)	0.197 (1.864)	0.310(5.927)
Fatigue*						
8.46 ± 5.30 (5.26 to 11.66)	7.31 ± 5.81 (3.80 to 10.82)	5.69 ± 3.17 (3.78 to 7.61)	5.31 ± 3.28 (3.33 to 7.29)	0.165 (2.182)	0.149 (2.379)	0.065 (4.115)
**Confusion**						
7.31 ± 4.35 (4.68 to 9.93)	6. 15 ± 4.49 (3.44 to 8.87)	6.31 ± 4.39 (3.66 to 8.96)	5.08 ± 4.13 (2.58 to 7.57)	0.856 (0.034)	0.092 (3.346)	0.826 (0.381)

Values are expressed in mean ± SD (CI95%). ^#^ Difference between moments (Time effect—ANOVA); * Difference between menstrual phases (Condition effect—ANOVA).

**Figure 1:**
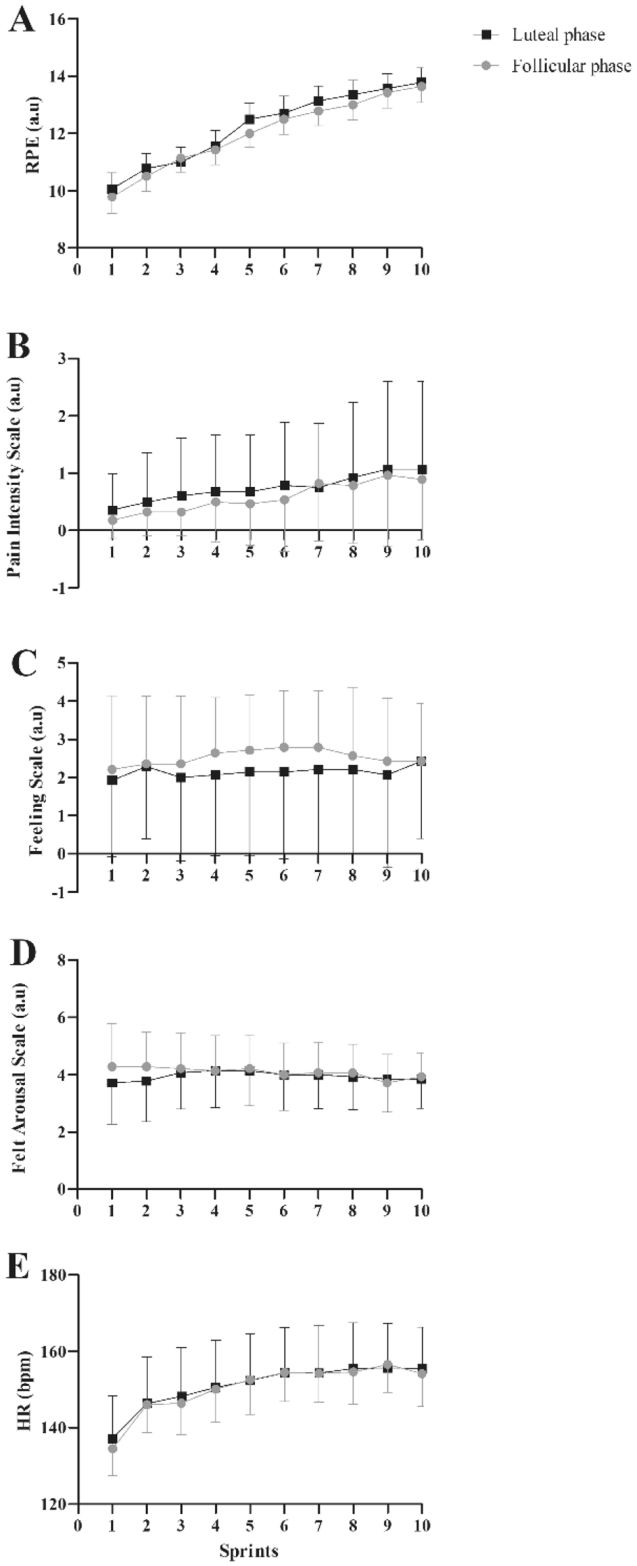
(**A)** Comparison between RPE during HIIE sprints of luteal and follicular phases. (**B)** Comparison between pain during HIIE sprints of luteal and follicular phases. (**C)** Comparison between feeling scale during HIIE sprints of luteal and follicular phases. (**D)** Comparison between arousal during HIIE sprints of luteal and follicular phases. (**E)** Comparison between heart rate during HIIE sprints of luteal and follicular phases. Data are expressed as mean ± SD (n = 14).

No significant differences in times were observed within luteal and follicular phases for RPE (*p* = 0.619; *F* = 0.652; *ƞ*^*2*^ = 0.048), pain (*p* = 0.395; *F* = 1.023; *ƞ*^*2*^ = 0.073), feeling (*p* = 0.158 *F* = 1.742; *ƞ*^*2*^ = 0.118), arousal (*p* = 0.324; *F* = 1.192; *ƞ*^*2*^ = 0.084), and HR (*p* = 0.840; *F* = 0.543; *ƞ*^*2*^: 0.043) during the 10 sprint runs in the HIIE (Fig. 1A–E). Likewise, [BLa] after HIIE was not different between luteal and follicular phases [2.17 ± 0.97 mmol∙L^−1^ (CI95% 1.61 to 2.74 mmol∙L^−1^) and 2.23 ± 1.02 mmol∙L^−1^ (CI95% 1.64 to 2.82 mmol∙L^−1^); *p* = 0.80)].

The time interference scores in the Stroop test were 11.06 ± 5.93 a.u. (CI95% 7.47 to 14.64 a.u.) and 9.71 ± 3.66 a.u. (CI95% 7.50 to 11.92 a.u.) for pre and post HIIE during the luteal phase, and 10.81 ± 5.70 a.u. (CI95% 7.37 to 14.26 a.u.) and 9.86 ± 6.51 a.u. (5.92 to 13.79 a.u.) for pre and post-HIIE during the follicular phase. There were no significant differences between times within conditions (*p* = 0.828; *F* = 0.049: *ƞ*^*2*^: 0.004), moments (*p* = 0.207; *F* = 1.780; *ƞ*^*2*^: 0.129), and menstrual phases (*p* = 0.970; *F* = 0.001; *ƞ*^*2*^: 0.00).

**Figure 2:**
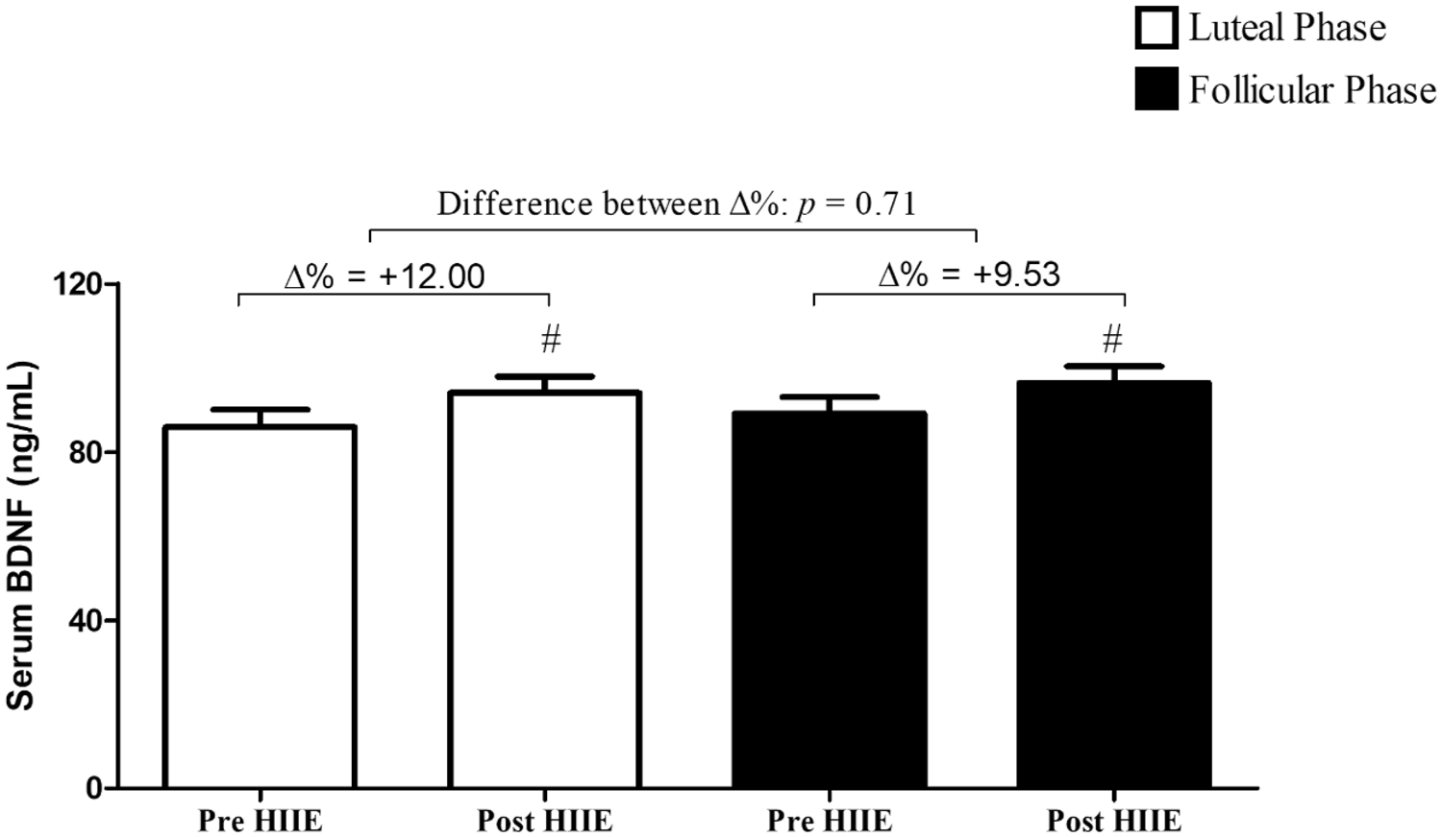
Comparison between BDNF measured at pre and post HIIE during luteal and follicular phases; # Difference between moments (Time effect—ANOVA). Data are expressed as mean ± SD (n = 14).

Figure 2 presents the BDNF results. No statistical differences were observed for BDNF absolute values among conditions (*p* = 0.87; *F* = 0.028; *ƞ*^*2*^ = 0.002) and between pre- and post-variation (∆%) in each phase. In addition, no differences were observed between menstrual phases (*p* = 0.35; *F* = 0.923; *ƞ*^*2*^= 0.066). On the other hand, statistical significance was observed between moments (*p* = 0.030; *F* = 6.057; *ƞ*^*2*^ = 0.318), showing that BDNF significantly increased after HIIE sessions for both conditions. Delta absolute difference (Δ) between pre and post HIIE during the luteal phase was 8.22 ± 17.43 ng/mL (CI95% − 1.85 to 18.28 ng/mL) and 7.29 ± 13.67 ng/mL (CI95% − 0.61 to 15.19 ng/mL). Similarly, for ∆%, the delta absolute difference was not different between conditions (*p* = 0.870).

**Figure 3:**
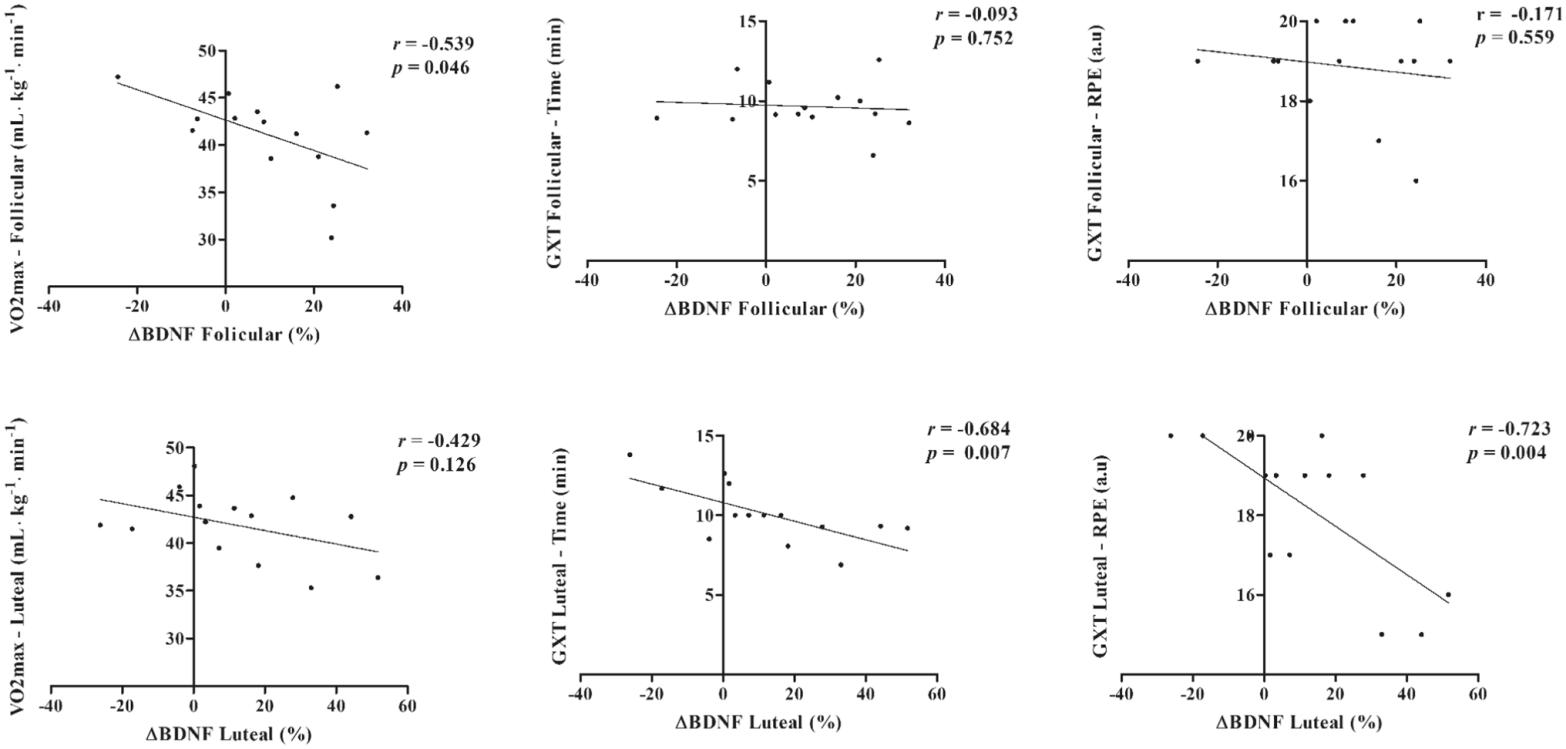
Correlation analysis between percentage BDNF changes and GXT variables, pre and post HIIE performed in luteal and follicular phases (n = 14).

Correlations between ∆% BDNF and $\dot{\text{V}}{{\text{O}}}_{\text{2max}}$, GXT performance, and RPE are expressed in Fig. 3. For follicular phase, only $\dot{\text{V}}{{\text{O}}}_{\text{2max}}$ demonstrated a significant correlation with ∆% BDNF (r =  − 0.539; *p* = 0.046), while in the luteal phase a significant correlation was observed between ∆% BDNF and GXT time to exhaustion (r =  − 0.684; *p* = 0.007) and RPE (r =  − 0.726; *p* = 0.004). In contrast, a non-significant correlation was observed between ∆% BDNF and $i\dot{\text{V}}{{\text{O}}}_{\text{2max}}$, PI-GXT, and [BLa]_peak_ for both phases (r ≥  − 0.51; *p* ≥ 0.06). In addition, no correlations were observed between ∆% BDNF and POMS dimensions of moods for luteal (r ≥  − 0.254; *p* ≥ 0.14) and follicular phases (r ≥  − 0.247; *p* ≥ 0.14), as well as with any subjective measurements and with peak [BLa] after HIIE for both phases (r ≥  − 0.378; *p* ≥ 0.25).

Finally, no significant correlations were found among ∆% BDNF and time interference score in the Stroop test for luteal (r ≥ 0.285; *p* ≥ 0.346) and follicular phases (r ≥  − 0.273; *p* ≥  0.368).

## Discussion

The current study aimed to evaluate the impact of HIIE performed in the luteal and follicular menstrual cycle phases on BDNF concentrations, cognitive function, mood, and exercise enjoyment in healthy women. Here, we showed for the first time that independently of menstrual phase, HIIE increases circulating BDNF levels. In addition, data from the current work indicate that HIIE mitigates tension, depression, and anger measured with POMS. On the other hand, we identified a negative relationship between the magnitude of peripheral BDNF changes (post–pre HIIE session) and $\dot{\text{V}}{{\text{O}}}_{\text{2max}}$ in the follicular phase. In the luteal phase an inverse relationship was observed among BDNF changes (post–pre HIIE session) with GTX-time and GTX-RPE. These data suggest that physical fitness status can exert an important impact on BDNF concentration after an HIIE session, mainly during the follicular phase.

The lack of differences in serum BDNF concentrations between luteal and follicular phases is in agreement with other authors^21^ who reported no differences between serum BDNF measured prior to exercise in luteal and follicular phases, or when compared with serum BDNF concentrations in men^21^. Despite this, the current results are in conflict with the findings of Begliuomini et al^19^, which may be explained by the collection moment in luteal and follicular phases, whether early, mid, or end (i.e., the blood samples were collected every 2 days), with the highest value of each phase being presented for comparison. In addition, the referred to study was conducted with a sample different from that used in other studies, given that the authors evaluated ovulatory, amenorrhoeic, and postmenopausal women, whereas in our investigation, as well in the El-Sayes et al^15^ study, only young women with a regular menstrual cycle were included.

However, to our best knowledge, no other studies have focused on evaluating the impact of HIIE on serum BDNF concentrations during different menstrual phases. Nevertheless, other works have identified the role of physical fitness status in modifying circulating BDNF concentration after exercise, especially in response to high intensity exercises^11,22^, providing evidence in agreement with our data, of higher serum BDNF concentrations after high intensity exercises.

Interestingly, our data indicated a strong relationship between circulating BDNF changes and physical fitness status in both menstrual cycle phases. Specifically, a negative correlation was observed between BDNF variation (Δ%) and $\dot{\text{V}}{{\text{O}}}_{\text{2max}}$ during the follicular phase; likewise, in the luteal phase, a negative correlation was observed between BDNF variation with GTX-time and GXT-RPE. Although the menstrual cycle per se does not affect physical performance^23^, some authors reported that hormonal fluctuations, such as estrogens (i.e., higher levels during follicular phase and lower levels in luteal phase) and progesterone (i.e., lower levels during follicular phase and higher levels during luteal phase) impact the inflammatory responses^24^. Specifically, estrogens have an important role in regulating immunocompetence (i.e., less anti-inflammatory gene activation during luteal phase)^24^. We consider that the latter condition might affect circulating BDNF levels in women. In fact, Weaver et al.^25^ found an association between BDNF and progesterone (r^2^ = 0.623, *p* = 0.01), suggesting that hormones may regulate BDNF concentration.

Moreover, the results suggested that BDNF concentration may be related to lactate accumulation, since it is inferred that muscle lactate is capable of crossing the blood–brain barrier and inducing an increase in BDNF via activation of Sirtuin1 deacetylase (SIRT1), with consequent increases in peroxisome proliferator-activated receptor gamma coactivator-1 alpha (PGC1α) and the secreted molecule FNDC5 (myokine gene that mediate BDNF release)^26^; however, there was no correlation between blood lactate concentration after HIIE and serum BDNF (Follicular phase: r^2^ =  − 0.24, *p* = 0.40; Luteal phase: r^2^ = 0.05; *p* = 0.85). One possible explanation for this result is the measurement of blood lactate concentration after HIIE, as the passive intervals between the sprints may underestimate the real concentration of the 10 high intensity sprints of HIIT^27^.

Besides the putative effect on BDNF, ovarian hormones can act on the hypothalamus and modify mood, pain perception, and exercise enjoyment^28^. Although no statistical differences were observed for these psychobehavioral parameters between menstrual phases, attenuation of subjective psychological parameters, such as tension, depression, and anger mood states (i.e., POMS) were found in both menstrual phases. It is worth indicating that mood variables were not correlated with BDNF concentrations after HIIE. These data suggest that acute mood changes induced by HIIE arise from other factors, such as the dopamine pathways^15^, since exercise regulation can increase dopamine receptor 2 and 4 sensitivity in the hippocampus and this may facilitate dopamine transmission to the ventral striatum and nucleus accumbens (brain areas related to exercise motivation and behavioral processes)^29^; however, this pathway was not measured in the present study. Further studies are justified to elucidate the possible impact of HIIE on the dopamine system.

Some precautions should be considered during interpretation of the presented data, mainly that even though all participants were required to have a regular menstrual cycle, the luteal and follicular phases were defined by self-reported control methods, without hormonal dosage^23^. In this way, it is possible that participants with symptoms of the menstrual cycle but without ovarian hormone fluctuations may have been included in the analysis, representing an important limitation of the current study.

Taken together, our data demonstrate that the menstrual cycle did not impact serum BDNF concentration, cognitive function, mood, and exercise enjoyment in healthy women after an acute HIIE session. However, acute HIIE increases serum BDNF concentration and attenuates tension, depression, and anger mood states independently of menstrual phase, suggesting the possibility of using HIIE as a strategy to attenuate the deleterious sensations occasioned by ovarian hormonal fluctuations. An important relationship was observed between BDNF changes and physical fitness status (i.e., $\dot{\text{V}}{{\text{O}}}_{\text{2max}}$) in the follicular phase, but not in the luteal phase. Finally, the study showed that physical fitness may be a relevant factor to regulate the magnitude of circulating BDNF changes during intermittent exercise and this information could be relevant for coaches and trainers.

## Methods

### Participants

For the current study, a minimum sample size of 4 subjects was calculated for a statistical power of 95% and alpha value of 0.05. Main outcomes were considered for the minimum sample size calculation. The sample size was calculated based on the findings of Begliuomini et al^19^, assuming an effect size (Cohen’s *d*) of 4.11. A total of 14 healthy, physically active female volunteers participated in the study (age: 24 ± 2 years; BMI: 22.79 ± 1.89 kg∙m^2^). Before beginning experimental procedures, participants were informed about the possible risks and benefits of the study and signed an informed written consent. All procedures were approved by the Sao Paulo State University Research Ethics Board (Protocol CAAE 92380318.5.0000.5402) and were conducted according to the Declaration of Helsinki. Additionally, participants were instructed not to perform any strenuous exercise for at least 72 h before each session and not to consume any ergogenic aid (i.e., caffeine) or drinks containing alcohol. All participants were required to have a regular menstrual cycle, attested by self-reported control methods, and not to have used any hormonal contraceptive methods at least 3 months prior to beginning the experiments. In addition, the participants could not use any anti-depressant or anxiety medications or have a medical diagnosis of depression or anxiety. This study was part of another project conducted between 2018-2019, entitled “Influence of the menstrual cycle phases on monocyte/macrophage polarization in sedentary and trained women” and herein, we present the cognitive function responses front exercise sessions.

### Experimental design

Prior to beginning the experimental procedures, the participants individually reported their menstrual cycle calendar for identification of the follicular (~ day 7 of menstrual cycle) and luteal phases (~ day 21 of menstrual cycle). After this, the participants visited the laboratory 4 times; 2 visits in the follicular phase and 2 in the luteal phase. In the first session of each phase the participants performed a graded exercise test (GXT) to measure maximal oxygen uptake ($\dot{\text{V}}{{\text{O}}}_{\text{2max}}$) and the peak intensity reached in the GXT (PI-GXT). In the second session of each phase, an HIIE session was performed at 90% of PI-GXT. An interval of 72 h was respected between the GXT and HIIE sessions (Supplemental Figure S1).

Ninety minutes before the HIIE sessions, a standard breakfast was offered to all participants (equivalent to 30% of individually estimated daily caloric expenditure) with macronutrient distribution of ≈35% lipids, ≈50% carbohydrates, and ≈15% proteins^30^. Before (90 min after breakfast) and immediately after the HIIE the inhibitory cognitive control was assessed using the Stroop color word test (SCWT) and mood state was assessed by the Profile of Mood States Questionnaire (POMS). Venous blood samples were collected before and immediately after the sessions for BDNF measurement. During every HIIE and after the end of each session, pleasure/displeasure, pain perception, arousal level, and rate of perceived exertion were assessed. After the HIIE, exercise enjoyment was measured using the Physical Activity Enjoyment Scale. In addition, 3 and 5 min after both GXT and HIIE sessions capillarized blood samples were collected from the ear lobe to determine peak blood lactate concentrations ([La]_peak_).

All exercise sessions were performed on a motorized treadmill (ATL, Inbramed, Porto Alegre, Brazil) with a fixed gradient at 1%^31^, using a safety belt to avoid accidental falls and induce maximal performance.

### Graded exercise test

The GXT started at 6.5 km∙h^−1^ with 1.5 km∙h^−1^ increments every 2 min until voluntary exhaustion^32^. Immediately after exhaustion, the participants remained in passive recovery for 5 min and performed a supramaximal exercise until exhaustion at an intensity of 105% of the PI-GXT (verification test)^33^. This supramaximal exercise was used as confirmation of $\dot{\text{V}}{{\text{O}}}_{\text{2max}}$. During GXT and supramaximal effort (i.e., confirmation of $\dot{\text{V}}{{\text{O}}}_{\text{2max}}$ ), the gas-exchange response was measured breath-by-breath using a gas analyzer (Quark CPET, COSMED, Rome, Italy) previously calibrated according to the manufacturer’s instructions. Heart rate (HR) was also measured using a wireless chest belt (HR Monitor; COSMED, Rome, Italy) synchronized with the gas analyzer. Data were smoothed every 10 points on Omnia Software 1.6.5 (COSMED, Rome, Italy) and interpolated each second with OriginPro 8.0 (OriginLab Corporation, Northampton, Massachusetts, USA).

The oxygen uptake of every completed stage during GXT was determined as the mean of the final 30 s, while the oxygen uptake during supramaximal exercise was determined considering the final 15 s of the effort^33^. $\dot{\text{V}}{{\text{O}}}_{\text{2max}}$ was assumed as the highest average of oxygen uptake when an oxygen uptake plateau was observed (difference < 2.1 ml∙kg^−1^∙min^−1^ between oxygen uptake in final two completed stages of GXT). When a plateau was not observed, the $\dot{\text{V}}{{\text{O}}}_{\text{2max}}$ was assumed as the highest value between the highest oxygen uptake value measured in the GXT and in the supramaximal effort, when the difference was < 2.1 ml∙kg^−1^∙min^−1^^34^. In addition, the $i\dot{\text{V}}{{\text{O}}}_{\text{2max}}$ was assumed as the lowest GXT intensity at which $\dot{\text{V}}{{\text{O}}}_{\text{2max}}$ was reached. If no $\dot{\text{V}}{{\text{O}}}_{\text{2max}}$ criteria were reached, the GXT session was repeated after at least 48 h.

### High-intensity intermittent exercise

Before HIIE, the participants performed 5 min of warm-up at 40% of $i\dot{\text{V}}{{\text{O}}}_{\text{2max}}$. The HIIE sessions consisted of 10 runs of 1 min at 90% of PI-GXT (upper 85% of maximal heart rate) with 1 min of passive recovery^35^, as recommended by Gibala, Gillen and Percival^36^ to generate adaptations from training (i.e., target intensity is between 80 and 100% of maximal HR). HR was measured beat-by-beat during all HIIE (Polar V800, Kempele, Finland). In addition, all HIIE were performed in the same period of the day to minimize the effects of the circadian cycle (between 7:00 a.m. and 12:00 p.m.), with controlled room temperature.

### Stroop color and word test

The Stroop Test was composed of three sets, each made up of fifty components and a task^37^. The set components could be a word or a color, using pink, blue, and green in accordance with a Brazilian validated Stroop Test version^38^. The first set was formed by fifty names of colors written in black ink and the second included fifty colorful blocks. In the third set, the names of the colors were printed with an incongruous ink color. The sets were denominated word (W), color (C), and color word (CW) respectively. In both the word and color sets, the task was to point to the components, while in the color word set, the task was to name the ink color, ignoring the word written. In all sets, the volunteers were encouraged to complete the task as quickly as possible and the time demanded to complete each task was measured using a chronometer. Moreover, the answers were recorded with a voice recorder. Subsequently, the time interference score (Ti) was calculated using Eq. (1)^39^.

1 $${\text{Ti}} = {\text{ CWT}} - \left[ {\frac{{\left( {{\text{WT}} - {\text{CT}}} \right)}}{2}} \right]$$

where CWT, WT, and CT refer to time to complete the task in color word, word, and color trials respectively.

### Subjective perception measurements

All participants were familiarized with the scales and questionnaires before the HIIE sessions. Mood state was assessed using the profile of mood states questionnaire (POMS)^40^. The scores of tension, depression, anger, vigor, fatigue, and confusion were measured according to Raglin and Morgan^40^. Pleasure/displeasure was assessed using the Feeling Scale^41^, leg pain perception using the Pain Intensity Scale (0 to 10)^42^, arousal level using the Felt Arousal Scale (0 to 6)^43^, and rate of perceived exertion (RPE) using the Borg Scale (6–20)^44^. Enjoyment was assessed after HIIE using the original 18 items of the Physical Activity Enjoyment Scale (PACES) with 7 point bipolar ratings^45,46^. Values of each item were summed, leading to a minimum score of 18 points and a maximum score of 126 points. Higher scores evidenced higher exercise enjoyment^46^.

### Blood sample analysis

Venous blood samples were collected from the antecubital vein (~ 10 mL) and immediately allocated into vacutainer tubes containing ethylenediaminetetraacetic acid (EDTA) for plasma separation and into dry vacutainer tubes for serum separation. Considering the variable specificities, only serum samples were used in the present study to quantify BDNF concentrations. The tubes were refrigerated for 1 h before centrifugation for 15 min at 3000 rpm and 4 °C (Centrifuge 5430 R, Eppendorf, Hamburg, Germany) for serum separation as previously described^10^. Next, the serum content was stored at − 20 °C until frozen and stored at − 80 °C until analysis. The serum BDNF concentration was analyzed using an enzyme-linked immunosorbent assay (ELISA) (R&D System, Minneapolis, MN, USA). The assay was performed following the manufacturer´s guidelines. Sensitivity of the enzymatic kit was 23.4–1500 pg/mL with an intra-assay coefficient of variation (CV) of 1.3%.

### Lactate determination

Capillary blood was collected from the earlobe (25μL) for measurement of blood lactate concentration [BLa]. Blood was collected using heparinized capillaries and stored at − 20 °C in microtubes containing 50μL of 1% sodium fluoride. The capillary blood samples were analyzed using a biochemical analyzer (YSI 2900; Yellow Spring Instruments, Yellow Spring, Ohio, USA, EUA), with an equipment error of ± 2%, according to the manufacturer’s information.

### Statistical analysis

Data are presented as mean ± SD and 95% confidence intervals (CI95%). For metabolic values the percentage variation (Δ%) between menstrual phases and moments (pre and post HIIE) was calculated assuming Luteal phase and pre exercise as 100%. The Shapiro–Wilk test was used to verify the normal distribution of data. For comparison between groups (i.e., menstrual cycle phase) and moments (i.e., pre and post HIIE) a two-way analysis of variance for repeated measures (two-way ANOVA) was used. In addition, Mauchly’s sphericity test was applied, and sphericity was assumed to be violated when the F-test was significant. In case of sphericity violation, the Greenhouse-Geiser Epsilon correction was used. Effect sizes for the ANOVA were calculated using partial eta squared (η^2^) for group, time, and interaction. The T-Test was used to compare the Δ% between phases and between moments. The Spearman correlation test was used to verify possible associations between BDNF, subjective perception measurements, and the Stroop test, while Pearson’s correlations were used to verify the possible association between BDNF and blood lactate concentration. In all cases, the significance level was ≤ 5% (*p* ≤ 0.05) and the data were analyzed using the Statistical Package for Social Sciences 22.0 (*SPSS* Inc. Chicago. IL.USA).

## Supplementary Information

Supplementary Information.
