# Supplementary material for: Peripheral BDNF and psycho-behavioral aspects are positively modulated by high-intensity intermittent exercise and fitness in healthy women
Source: Sci Rep. 2021 Feb 18;11:4113. doi: 10.1038/s41598-021-83072-9 (PMC7893166; doi:10.1038/s41598-021-83072-9)
Supplement: Supplementary file 1 — Supplementary Information. [file 41598_2021_83072_MOESM1_ESM.docx]

**Peripheral BDNF and** **psycho-behavioral aspects are positively modulated by high-intensity intermittent exercise and fitness in healthy women**

Rodrigo Araujo Bonetti de Poli, Vithor Hugo Fialho Lopes, Fábio dos Santos Lira, Alessandro Moura Zagatto, Alberto Jimenez Maldonado, Barbara Moura Antunes.

**Supplemental Table S1.** GXT parameters comparation between luteal and follicular phases

|  | **Luteal** |  | **Follicular** |  | **∆%** |  | ***p*** |
| --- | --- | --- | --- | --- | --- | --- | --- |
| $\dot{\text{V}}\text{O}_{\text{2max}}$ (mL∙kg^-1^∙min^-1^) | 41.91 ± 3.75 (39.65 to 44.19) |  | 41.30 ± 4.80 (38.40 to 44.20) |  | -1.94 |  | 0.31 |
| $i\dot{\text{V}}\text{O}_{\text{2max}}$ (km∙h^-1^) | 11.8 ± 1.3 (11.0 to 12.6) |  | 11.6 ± 1.1 (10.9 to 12.3) |  | -1.11 |  | 0.42 |
| PI-GXT (km∙h^-1^) | 12.3 ± 1.2 (11.5 to 13.1) |  | 12.2 ± 1.1 (11.5 to 12.9) |  | -0.32 |  | 0.67 |
| HR_max_ (bpm) | 189 ± 9 (183 to 194) |  | 191 ± 8 (186 to 196) |  | +1.12 |  | 0.20 |
| RER (a.u) | 1.10 ± 0.07 (1.06 to 1.15) |  | 1.11 ± 0.06 (1.07 to 1.14) |  | +1.16 |  | 0.77 |
| RPE (a.u) | 18 ± 2 (17 to 19) |  | 19 ± 1 (18 to 19) |  | +4.40 |  | 0.21 |
| [La]_peak_ (mmol∙L^-1^) | 8.70 ± 2.36 (7.27 to 10.12) |  | 8.72 ± 2.31 (7.33 to 10.12) |  | +3.48 |  | 0.96 |
| GXT time to exhaustion (min) | 9.97 ± 1.89 (8.83 to 11.12) |  | 9.70 ± 1.55 (8.76 to 10.64) |  | -3.63 |  | 0.13 |

Values are mean±SD (CI95%). $\dot{\text{V}}\text{O}_{\text{2max}}$ = maximal oxygen uptake; PI-GXT = peak intensity reached in graded exercise test; $i\dot{\text{V}}\text{O}_{\text{2peak}}$ = peak of intensity reached in GXT; HR_max_ = maximal heart rate reached in GXT; RER = Respiratory exchange ratio; RPE = Rating of perceived exertion; [La]_peak_ = peak blood of lactate concentration; ∆% = percentage difference between luteal and follicular phases.


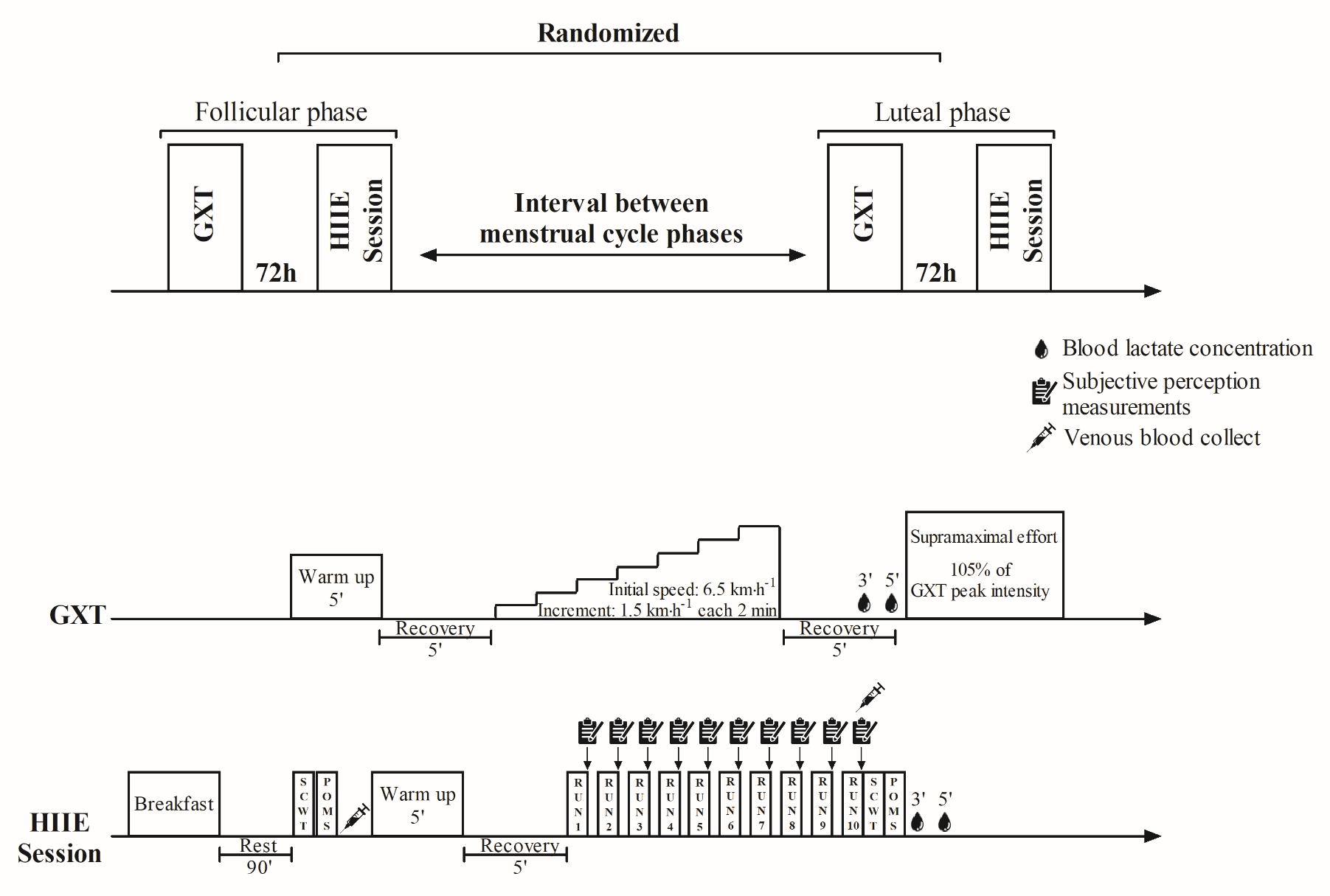


**Supplemental Figure S1.** Experimental Design of Study. SCWT = Stroop color word test; POMS = Profile of mood states questionnaire.
